# A genetically anchored physical framework for *Theobroma cacao *cv. Matina 1-6

**DOI:** 10.1186/1471-2164-12-413

**Published:** 2011-08-16

**Authors:** Christopher A Saski, Frank A Feltus, Margaret E Staton, Barbara P Blackmon, Stephen P Ficklin, David N Kuhn, Raymond J Schnell, Howard Shapiro, Juan Carlos Motamayor

**Affiliations:** 1 Clemson University Genomics Institute, Clemson University, 51 New Cherry Street, Clemson, SC 29634, USA; 2 Department of Genetics and Biochemistry, Clemson University, 51 New Cherry Street, Clemson, SC 29634, USA; 3 Subtropical Horticulture Research Station, USDA-ARS, 13601 Old Culter Road, Miami, FL 33158, USA; 4 Mars, Inc., 800 High St., Hackettstown, NJ 07840, USA

## Abstract

**Background:**

The fermented dried seeds of *Theobroma cacao *(cacao tree) are the main ingredient in chocolate. World cocoa production was estimated to be 3 million tons in 2010 with an annual estimated average growth rate of 2.2%. The cacao bean production industry is currently under threat from a rise in fungal diseases including black pod, frosty pod, and witches' broom. In order to address these issues, genome-sequencing efforts have been initiated recently to facilitate identification of genetic markers and genes that could be utilized to accelerate the release of robust *T. cacao *cultivars. However, problems inherent with assembly and resolution of distal regions of complex eukaryotic genomes, such as gaps, chimeric joins, and unresolvable repeat-induced compressions, have been unavoidably encountered with the sequencing strategies selected.

**Results:**

Here, we describe the construction of a BAC-based integrated genetic-physical map of the *T. cacao *cultivar Matina 1-6 which is designed to augment and enhance these sequencing efforts. Three BAC libraries, each comprised of 10× coverage, were constructed and fingerprinted. 230 genetic markers from a high-resolution genetic recombination map and 96 Arabidopsis-derived conserved ortholog set (COS) II markers were anchored using pooled overgo hybridization. A dense tile path consisting of 29,383 BACs was selected and end-sequenced. The physical map consists of 154 contigs and 4,268 singletons. Forty-nine contigs are genetically anchored and ordered to chromosomes for a total span of 307.2 Mbp. The unanchored contigs (105) span 67.4 Mbp and therefore the estimated genome size of *T. cacao *is 374.6 Mbp. A comparative analysis with *A. thaliana, V. vinifera*, and *P. trichocarpa *suggests that comparisons of the genome assemblies of these distantly related species could provide insights into genome structure, evolutionary history, conservation of functional sites, and improvements in physical map assembly. A comparison between the two *T. cacao *cultivars Matina 1-6 and Criollo indicates a high degree of collinearity in their genomes, yet rearrangements were also observed.

**Conclusions:**

The results presented in this study are a stand-alone resource for functional exploitation and enhancement of *Theobroma cacao *but are also expected to complement and augment ongoing genome-sequencing efforts. This resource will serve as a template for refinement of the *T. cacao *genome through gap-filling, targeted re-sequencing, and resolution of repetitive DNA arrays.

## Background

*Theobroma cacao *(cacao tree) is the source of the world's cocoa butter and cocoa powder, key ingredients in chocolate. *T. cacao *is a short, tropical tree that is grown in multiple countries including Côte d'Ivoire, Ghana, Indonesia, Nigeria, Brazil, Cameroon, Ecuador, Colombia, Mexico, and Papua New Guinea where cacao beans are an important cash crop. World cocoa production was estimated to be 3 million tons in 2010 and had an annual estimated average growth rate of 2.2% from 1998 to 2010 (http://www.worldcocoafoundation.org/learn-about-cocoa/cocoa-facts-and-figures.html). Cacao bean production is currently under threat from several sources including a rise in the incidence of fungal diseases including black pod, frosty pod, and witches' broom [1,2]. In order to address these issues, multiple genetic and genomic efforts have been initiated in the last decade to identify genetic markers and genes that could be utilized to accelerate the release of robust *T. cacao *cultivars [3-10]. These efforts have recently culminated in whole-genome shotgun assemblies of the genomes of two *T. cacao *cultivars: Criollo [11] and Matina 1-6 [12]. These genome sequences will greatly assist *T. cacao *breeding efforts [10] as well as contribute to our basic knowledge of tree and dicot biology through comparisons with a growing collection of genome sequences from trees and dicotyledonous plants.

There are primarily two approaches to sequence a genome: the BAC-by-BAC approach where libraries of clones with large inserts (e.g. BACs) are randomly sequenced and ordered relative to a minimum tile path (MTP) such as has been used to sequence the genomes of rice and maize [13,14], or the whole- genome shotgun (WGS) sequencing of genomic DNA, carried out without cloning, from genomic libraries with multiple insert sizes as implemented for Western poplar, grapevine, and sorghum genomes [15-17]. In the BAC-by-BAC approach, the MTP is obtained through the construction of a physical map via DNA fingerprinting: each BAC clone is digested with restriction enzymes [18-20] and contigs are generated based upon shared DNA signatures. DNA signatures, comprised of a BAC's repertoire of fragment sizes, are determined by algorithms incorporated into software such as Fingerprinted Contigs (FPC) [18,21]. FPC-based physical maps can be validated and improved by incorporating information obtained by hybridizing BAC clones with specific probes which can include probes that have been genetically mapped; the ordering of contigs on physical maps can thus be facilitated by using genetically derived mapping information [22]. Furthermore, BAC ends can be sequenced and serve as well-spaced markers for determining the accuracy of shotgun read assemblies. Using a strategy that integrates physical mapping and genetic mapping reduces the number of chimeric contigs and increases overall confidence in the final assembly (whether BAC-by-BAC or WGS). In any strategy, it is important to have a high quality reference assembly available for annotation and re-sequencing endeavours.

In this study, we describe the construction of a BAC-based integrated genetic-physical map of the genome of *T. cacao *cv. Matina 1-6 (estimated genome size = 374 Mbp). We also demonstrate the utility of the map by comparing it with other plant genomes. This map serves as an important reference for *T. cacao *genomes being sequenced; it can be used to establish the accuracy of those genome sequences prior to their use for applications such as SNP discovery, RNAseq, ChiPseq, and other techniques.

## Results

### Physical map construction

*T. cacao *genomic DNA was first cleaved into large fragments for cloning into vectors that could accommodate large inserts. Three different restriction endonucleases, *Hin*dIII, *Mbo*I, and *Eco*RI, were utilized to partially digest genomic DNA samples which were then used to construct three *T. cacao *BAC libraries (TCC_Ba, TCC_Bb, TCC_Bc, respectively) using methods that have been previously described [23]. These libraries were then used to construct the physical map. 36,864 clones were arrayed for each of the three complementary BAC libraries, which we estimate provides 10× genome coverage and, therefore, these libraries collectively represent approximately 30 *T. cacao *genome equivalents. The average insert size was 138 kb and the clones are arrayed in 288, 384-well microtiter plates as summarized in Table 1.

**Table 1:** *T. cacao *BAC library overview

Library	Restriction enzyme	BAC vector	No. clones	Average insert size (kb)	Insert range (kb)	Genome coverage*	HICF fragments (avg)	Control clones	Successful fingerprints
TC_Bba	HindIII	pIndigoBAC536	36864	145	90-185	10×	120.2	768	31864

TC_BBb	EcoRI	pIndigoBAC536	36864	120	40-160	10×	102.1	768	32218

TC_BBc	MboI	pIndigoBAC536	36864	140	80-180	10×	120.4	768	31304

*Assumes 440 Mbp genome size.

In order to determine BAC order and orientation, we characterized all BACs in the libraries using high resolution restriction band fingerprinting. Using previously published methods [24], a total of 108,288 BAC clones from all three BAC libraries were subjected to high information content fingerprinting (HICF) after addition of control clones in the E07 and H12 wells of the 96-well offset to maintain data uniformity. Data comprising fragment sizes were captured by capillary electrophoresis [24]. After removal of clones containing less than 20 fragments or greater than 220 fragments and empty vectors, 95,386 clones (88% of the original total), with an average of 114.2 fragments per clone, were successfully assigned a digitized fingerprint (Table 1) to use for assembly carried out using the FPC software [25].

To obtain a high-quality commensurate build, fingerprints from all three BAC libraries were combined for contig assembly using a stringent cutoff of 1 e^-80 ^and a tolerance of 3. This base assembly resulted in 445 contigs harbouring 86,590 clones and 8,806 singletons. The DQer function of FPC was run to identify and break up contigs comprised of greater than 10% questionable clones (a sign of potential false joins). The Singles-to-Ends function and the Ends-to-Ends function were run at a final cutoff of 1 e-^50 ^using automatic merges to join singletons to contig ends and contigs to contigs, respectively. Additionally, manual curation of the physical map was performed based on results collected from the integration of the genetic recombination map and synteny mapping (described below) at a cutoff as low as 1 e^-25^. Assuming a cumulative average insert size of 138 kb and an estimated *T. cacao *genome size of 440 Mbp, the consensus band (CB) estimation equates to an average of 1,210 bp per band.

**Figure 1:**
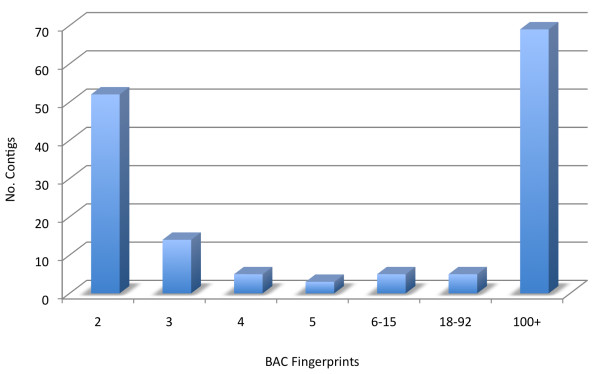
**Number of BAC fingerprints per FPC contig in the *T. cacao *physical map**. The major FPC contigs are built with more than 100 BACs per contig.

The final *T. cacao *physical map totaled 154 contigs containing 91,117 BACs (96%) and 4,268 singletons (4%) (Table 2). Of the 154 contigs, 74 contigs are comprised of fewer than 5 BACs, 5 contigs are comprised of between 6 and 15 BACs, 5 others of between 18 and 92, and 69 are comprised of greater than 100 BACs per contig (Figure 1). Size estimations of consensus bands were converted to base pairs in the summary of the *T. cacao *physical map assembly and for estimates of contig lengths (Tables 2 and 3).

**Table 2:** *T. cacao *physical map overview

Category	Value
No. BACs in FPC	95,386
No. BACs in contigs	91,117
Contigs	154
Singletons	4,268
Contigs, Anchored	49
No. BACs in Anchored Contigs	79,298
Contigs, Unanchored	105
No. BACs in Unanchored Contigs	11,190
Contig Len, Anchored (Mbp)	307.2
Contig Len, Unanchored (Mbp)	67.4
FPC Genome Size (Mbp)	374.7
% Genome Anchored (bp)	82.0%

**Table 3:** Pseudomolecule Statistics

Pseudomolecule	^**1,2**^**Length(bp)**
Pseudo1	38,543,146
Pseudo2	56,067,851
Pseudo3	31,717,810
Pseudo4	30,918,000
Pseudo5	37,968,944
Pseudo6	23,261,314
Pseudo7	20,970,042
Pseudo8	17,682,746
Pseudo9	38,614,810
Pseudo10	21,003,454
ALL	316,748,117

*^1^With 0.25 Mbp gaps between contigs*.

^2^1 CB = 1210 bp

### Integration of genetic markers and sequence tagged sites (STS) onto the physical map

**Figure 2:**
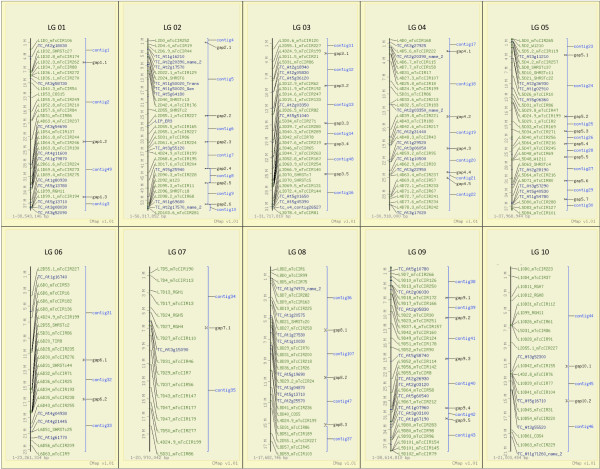
**A cMAP view of the 49 anchored *T. cacao *BAC contigs arranged and oriented as pseudomolecules according to the integrated genetic map spanning the 10 chromosomes**. BAC contigs are indicated with blue brackets, associated recombination markers are labeled in green, and AtCOSII [30] converted to TcCOSII markers are labeled in blue.

To assimilate genetic and physical maps and integrate conserved ortholog set (COS) sequences onto the *T. cacao *physical framework, overgo probes were designed from genetically mapped simple-sequence repeat markers (SSR) originating from EST sequences [5,26] and [6] COSII sequences derived from *Arabidopsis thaliana *[27], respectively. The remaining 95 COSII sequences were placed onto the physical map and their linkage groups inferred by contig placement. Overgo probes were anchored to the physical map using a 3-dimensional pooled hybridization approach following the method of Fang et al. [28]. Briefly, overgo probes were pooled using a pooling strategy in which 125 probes were hybridized to the three *T. cacao *BAC libraries. Based on single location integration, 96% of the markers were accurately placed on the physical map and these markers were used to anchor and orient 49 contigs as 10 pseudomolecules (Table 3). Where ordered contig ends fail to overlap, an arbitrary 250 kb addition was made and annotated as gaps between contigs and is visualized using the CMAP comparative map viewer (Figure 2; [29]); this gap addition was not calculated as part of any physical map statistics. A framework file was created in FPC to anchor BAC contigs to chromosomes using results obtained from integration of 230 genetic recombination markers [30] (Additional file 1: Table S1, Additional file 2: Table S2). The framework function of FPC was used to anchor BAC contigs to chromosomes, as well as order and renumber them, which resulted in 49 contigs assigned, ordered, and oriented to the ten *T. cacao *linkage groups; 105 contigs remained unanchored, most of which contain a small number of BACs. Specific contigs (anchored and unanchored) are described in Additional file 3: Table S3 and Additional file 4: Table S4.

### Dense minimum tile path (MTP) selection for BAC-end sequencing

BAC-end sequences (BES) corresponding to a fingerprint in the physical map serve as long-range, paired sequence anchor points which can be used to facilitate alignments to other genomes and integration of draft sequence contigs. Ideally, a pair of BAC-end sequences assigned to each BAC in the physical map will provide a robust array of Sequence Tagged Sites (STS) for draft genome anchoring, genome exploitation, etc. However, the *T. cacao *physical map assembled into a very few, long contigs built from nearly 30× BAC coverage; therefore, we determined a BES for every clone was not necessary but rather an end sequence every 7-8 kb. For the *T. cacao *physical map, the median number of Consensus Band (CB) units per BAC was 120 CBs, the average BAC insert size was 138 kb, and the desired distance in bp between sequence anchor points was 8 kb. The resulting n-value was approximately 7 CB units and 29,383 BACs with an end approximately every 7CB units apart were selected for BAC-end sequencing. A total of 58,766 sequencing reactions were performed that included both ends, 52,966 (90%) of which were successfully trimmed and a total of 49,984 of those were part of a successful pair. This moderately dense array of BAC-end sequences will serve as sequence anchor points for comparative genomics and long-range sequence pairs for draft genome sequence integration.

### Alignment of the *T. cacao *physical map with *V. vinifera, P. trichocarpa*, and *A. thaliana *genomes

As more genomes are sequenced, comparative genomics is becoming a more readily applicable approach to studying genomic architecture, gene function, and genome evolution across species. Recently, Argout et al. [11] published findings into the paleohistory of *T. cacao *(cv. Criollo) by looking at orthologous genes between *T. cacao *(cv. Criollo) chromosomes and *Vitis vinifera, Arabidopsis thaliana, Populus trichoptera, Glycine max*, and *Circa papaya*. We used a similar synteny-based approach to gain insight into *T. cacao *(cv. Matina 1-6) genome structure and evolutionary history; we compared the genetically integrated *T. cacao *physical map to the *V. vinifera, P. trichocarpa*, and *A. thaliana *genomes using 52,966 BAC-end sequence anchor points tied to 49 genetically anchored BAC contigs, 230 genetically mapped framework markers, 6 mapped AtCOSII markers, and 95 AtCOSII anchored markers for each of the alignments below. The alignments and visualizations were performed with the Symap software [31,32]. A detailed review of the synteny computing algorithm used can be found in Soderlund et al. [31]; briefly, the BES and marker sequences were filtered for repeats aligned to the corresponding genomes. A total of 13,807 BES hits anchored to 60 fingerprint contigs, covering approximately 73% (321.5 Mbp) of the *V. vinifera *genome (Table 4; Additional file 5: Table S5), were aligned between the *T. cacao *physical map and the *V. vinifera *genome. The average percent identity (% identity) of the alignment of the BES-associated contigs ranged from 74% to 100%. A total of 101 synteny blocks were identified, 27 of which were less than 1 Mbp long, 43 were between 1 and 3 Mbp in length, and 31 were greater than 3 Mbp (Table 5). With *P. trichocarpa*, 10,731 BES hits and 57 marker sequences were anchored to the 63 contigs that could be aligned, covering approximately 78% (403.5 Mbp) of the *P. trichocarpa *genome (Table 4; Additional file 6: Table S6). The percent identity of the BES alignments with *P. trichocarpa *ranged from 75% to 100%. A total of 187 synteny blocks were identified, 87 of which were less than 1 Mbp, another 61 were between 1 and 3 Mbp, and 39 were greater than 3 Mbp (Table 5). Approximately 44% (75.9 Mbp) of the *A. thaliana *genome was covered (Table 4; Additional file 7: Table S7; Table 5) as a result of 5,332 BES hits and 59 marker sequences anchored to 48 contigs. It is important to note here that even though 96 COSII sequences were used as anchor points, only 59 of them were considered a homologous match in *A. thaliana*; the unmatched COSII sequences likely were flanked by non-homologous BES that did not meet the criteria set to be considered a syntenic region. The percent identity of the sequence matches between *A. thaliana *and *T. cacao *ranged from 75% to 95% and a total of 68 synteny blocks were identified, 50 of which were less than 1 Mbp, 14 ranged between 1 and 3 Mbp, and 4 were greater than 3 Mbp (Table 5). Alignments of our physical map to these three genomes are consistent with *T. cacao's *closest relative being *P. trichocarpa*, followed by *V. vinifera *and then *A. thaliana*, the most distant relative of the three. Structural details are discussed below.

**Table 4:** Synteny coverage between *T. cacao *(Matina 1-6) physical map and reference genome assemblies

Reference Genome	Coverage Genome	Coverage	Double-coverage*
*V. vinifera*	*T. cacao*	87%	56%

*V. vinifera*	*V. vinifera*	73%	27%

*P. trichoptera*	*T. cacao*	91%	78%

*P. trichoptera*	*P. trichoptera*	78%	24%

*A. thaliana*	*T. cacao*	71%	35%

*A. thaliana*	*A. thaliana*	44%	13%

*T. cacao (Criollo)*	*T. cacao (Matina 1-6)*	97%	72%

*T. cacao (Criollo)*	*T. cacao (Criollo)*	65%	25%

*Double coverage is the fraction of sequence that is covered by more than one synteny block.

**Table 5:** Synteny blocks between *T. cacao *(Matina 1-6) physical map and reference genome assemblies

Reference Genome	^**1**^**Anchors**	^**1**^**Block Hits**	^**1**^**One block**	^**1**^**Two blocks**	Blocks (< 1 Mbp)	Blocks (1 Mbp - 3 Mbp)	Blocks (> 3 Mbp)
***V. vinifera***	13,807 (0)	3,266 (0)	2712 (0)	277 (0)	27	43	31
***P. trichoptera***	10,788 (57)	5,535 (15)	2,517 (7)	1,509 (4)	87	61	39
***A. thaliana***	5,391 (59)	697 (1)	583 (1)	61 (4)	50	14	4
***T. cacao (Criollo)***	37,504 (194)	26,739 (130)	21,765 (108)	2,487 (11)	56	25	31

^1^Total anchors (BES + genetic markers) with genetic marker subset in parentheses.

**Figure 3:**
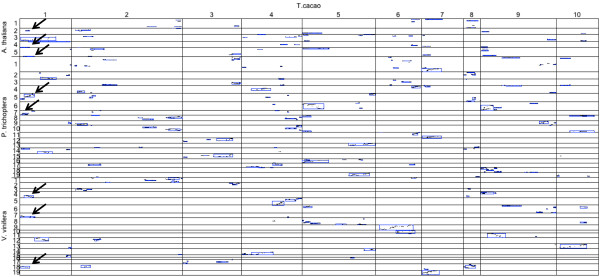
**A SyMAP whole-genome dot plot with the *T. cacao *physical map pseudomolecules as reference compared to *A. thaliana, P. trichocarpa, and V. vinifera *genomes**. Blue rectangles indicate synteny blocks and bold arrows highlight segmental duplications of *T. cacao *chromosome 1 across the three species.

**Figure 4:**
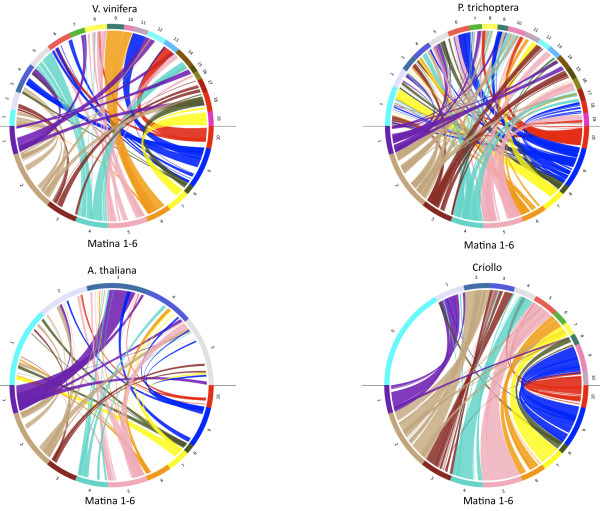
**Circos plots of the alignments between *T. cacao *cv**. Matina 1-6 versus *V. vinifera, P. trichocarpa, A. thaliana, and T. cacao *cv. Criollo. Colored ribbons indicate matches between the *T.cacao *cv. Matina 1-6 pseudomolecules and the other respective genomes. Potential chromosome fusion events are illustrated when *T. cacao *chromosomes match two or more locations on respective genomes.

In order to visualize synteny relationships, whole-genome dot plots and circos plots [32] were created to visualize genome structure and collinearity between *T. cacao *and the three other genomes (Figures 3 and 4). As described above, the most syntenic blocks were identified between *T. cacao *and *P. trichocarpa *followed by those with *V. vinifera*, and then *A. thaliana*. The longest stretch of collinearity is between *T. cacao *and *P. trichocarpa *and spans 26.7 Mbp; the longest span with *V. vinifera *is 18.9 Mbp and with *A. thaliana *is 5 Mbp. There are several duplications of *T. cacao *chromosomal segments that can be visualized within the three target genomes. For example, regions of *T. cacao *chromosome 1 appear to be duplicated in *A. thaliana, P. trichocarpa*, and *V. vinifera *(Figures 3 and 4). The longest duplicated synteny block between *T. cacao *and *A. thaliana *is a duplicated segment on *T. cacao *chromosome 5 that spans 13,952 CB units or approximately 16.6 Mbp and aligns to chromosomes 5 and 2 of *A. thaliana*. A more distal segment of *T. cacao *chromosome 5 is duplicated in *P. trichocarpa *on chromosomes 13 and 18 and is estimated to be approximately 13.8 Mbp (Figures 3 and 4). A segment of *T. cacao *chromosome 10 is present in three copies in *V. vinifera *(located on chromosomes 6, 8, and 12) that span a total of 3,054 CB units or approximately 3.7 Mbp. Argout et al. proposed an evolutionary scenario that suggests the 10-chromosome structure of *T. cacao *was formed from an intermediate ancestor with 21 chromosomes through eleven chromosome fusion events [11]. We observed evidence of chromosomal fusion events as well through comparisons of the *T. cacao *physical map to *V. vinifera, P.trichoptera*, and *A. thaliana *as shown in the circos plots (Figure 4). For example, *P. trichocarpa*chromosomes 2, 5, and 14 may have fused to form *T. cacao *(Matina 1-6) chromosome 1. *V. vinifera *chromosomes 4, 6, and 11 may have fused to form *T. cacao *chromosome 9 (Figure 4).

To determine if a physical map is sufficient for investigating ancestral paleo-polyploidy events, we looked at the detailed alignments between *T. cacao *and *V. vinifera *chromosomes. We observed that *V. vinifera *chromosomes 1, 14, and 17 align to *T. cacao *(Cv. Matina 1-6) chromosomes 2, 3, and 4; *V. vinifera *chromosomes 2, 12, 15, and 16 align to *T. cacao *(Cv. Matina 1-6) chromosomes 1, 3, and 5; *V. vinifera chromosomes *3, 4, 7, and 18 align to *T. cacao *chromosomes 1, 2, and 8; *V. vinifera chromosomes *4, 9, and 11 align to *T. cacao *6, 8, and 9; *V. vinifera *chromosomes 5, 7, and 14 align to *T. cacao *1, 4, and 5; *V. vinifera *chromosomes 6, 8, and 13 align to *T. cacao *5, 9, and 10; *V. vinifera *chromosomes 10, 12, and 19 align to *T. cacao *1, 6, and 7 (Figure 4). These observations are nearly identical to those of Argout et al. [11] and also confirm evidence of ancestral triplicated chromosome groups reported for *V. vinifera *[15]. These results suggest that a BAC-based physical map with relatively evenly spaced BAC-end sequence anchor points can have immediate utility, depending on the amount of collinearity that exists between it and other genomes of interest, for interrogating agriculturally important genes and gene families and elucidating evolutionary origins prior to the availability of a high quality reference genome.

### Alignment of the *T. cacao *cv. Matina 1-6 physical map with the *T. cacao *cv. Criollo genome assembly

After the *T. cacao *cv. Matina 1-6 physical map was constructed the *T. cacao *cv. Criollo genome assembly became available [11]. Alignment of the *T. cacao *cv. Matina 1-6 physical map with the *T. cacao *cv. Criollo genome assembly [11] identified a total of 37,310 BES matches and 194 marker sequences anchored to 87 contigs covering approximately 65% (318.3 Mbp) of the Criollo genome (Table 4; Additional file 8: Table S8). The average percent identity of the sequence matches ranged from 83% to 99%. A total of 112 synteny blocks were identified, 56 of which were less than 1 Mbp in length, 25 ranged from 1 to 3 Mbp, and 31 were longer than 3 Mbp (Table 5). Alignment of the genome sequences of the Matina 1-6 cultivar with those of the Criollo cultivar revealed a very close alignment, as expected, which validates the comparative genomic software and methodology.

**Figure 5:**
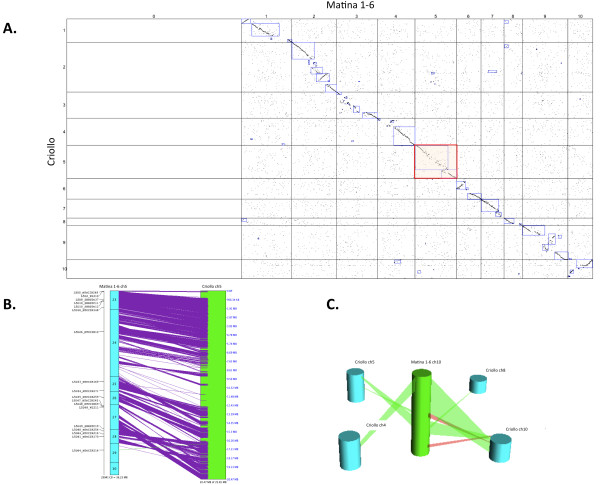
**Alignment of the *T. cacao *cv**. Matina 1-6 physical map to the *T. cacao *cv. Criollo draft genome sequence. **A**. A SyMAP dot plot alignment of the anchored contigs of the *T. cacao *cv. Matina 1-6 physical map and the *T. cacao *cv. Criollo genome assembly [11]. Purple boxes highlight synteny blocks. The red shaded box is a dot plot view of alignments detailed in 5B. **B**. A 2D view of *T. cacao *cv. Matina 1-6 chromosome 5 aligned to *T. cacao *cv. Criollo chromosome 5. Purple lines represent BES matches to respective locations. Markers from the *T. cacao *cv. Matina 1-6 chromosome 5 linkage group are designated on the left. **C**. A SyMAP 3D view illustrating duplications of regions of *T. cacao *cvv. Matina 1-6 pseudomolecule ten and matches for those regions on various *T. cacao *cv. Criollo chromosomes. Green shade indicates direct orientation and red shade is inverted orientation.

While alignment of the Matina 1-6 anchored physical map contigs to the Criollo genome sequence revealed long stretches of collinear sequence (Figure 4; Figure 5A), there were also many instances of rearrangements by either inversion or translocation and instances of duplication (Figure 5). Segments of Criollo chromosome 1 are duplicated on Matina 1-6 chromosomes 4, 8, and 9, for example. There are also segments of Criollo chromosome 4 and chromosome 5 that are duplicated on Matina 1-6 chromosome 10 and chromosomes 2 and 10, respectively. There are segments on Criollo chromosome 7 that are duplicated on Matina 1-6 chromosome 2. There is a segment of Criollo chromosome 8 that is duplicated on Matina 1-6 chromosome 1 and 2. An example of an inverted genomic segment resides near the telomere on Criollo chromosome 1 (Figure 5A) and another on chromosome 5 (Figure 5A and 5B). Two potential sequence translocations are located on Criollo chromosomes 1, 5, and 7, reside on chromosomes 4, and 2 in Matina 1-6, respectively, but further support is necessary to confirm these due to low resolution in these areas. As an example of using a comparative approach to elucidate the structure of the Matina 1-6 genome, Figure 5C illustrates duplicated sequences from Matina 1-6 linkage group 10 that match sequences on Criollo chromosomes 4, 5, 8, and 10. Figure 5B shows a 2D comparison of Matina 1-6 chromosome 10 and Criollo chromosome 10. These chromosomes are apparently highly similar in sequence but quite rearranged. The longest conserved segment of contiguous collinearity between the sequences of the two cultivars occurs on chromosome 2 and spans approximately 15.3 Mbp. The most non-congruent chromosome between the two cultivars is chromosome 3 and the most rearranged is chromosome 9. Investigating these structural differences could reveal the underlying biological mechanisms that directed these events from an evolutionary standpoint. Assembly accuracy of both the Matina 1-6 physical map and the Criollo draft assembly should also be verified.

### Integration of unanchored contigs using collinearity with other genomes

In an effort to improve the *T. cacao *cv. Matina 1-6 physical map, we examined structural similarities found in the genomes of related species that might suggest possible linkage group assignments for unanchored contigs in the map. Unincorporated Matina 1-6 FPC contigs were assessed for linkage group assignment based on anchoring to regions of the *V. vinifera, P. trichocarpa, A. thaliana*, and *T. cacao *cv. Criollo chromosomes where other *T. cacao *cv. Matina 1-6 anchored BAC contigs were aligned. These alignments resulted in linkage group predictions for 11 unanchored contigs (Additional file 9: Table S9). As more genomes are sequenced, a comparative approach to refining existing physical maps will become more effective.

## Discussion

### Overview of the cacao physical map

We constructed three BAC libraries together representing ~30× coverage of the *T. cacao *genome (publicly available via http://www.genome.clemson.edu) and used them to create the first whole-genome physical map for *T. cacao *cv. Matina 1-6 (publicly available via http://www.cacaogenomedb.org). This map is genetically anchored and enables cacao cultivar improvement through efforts such as positional cloning and region-specific analysis through sub-genome sequencing (companion paper). The map also aids in assembly of reference genomes, gap-filling, and independent assessment of assembly accuracy. And, in addition to protein-coding regions, BACs harbor genomic segments such as untranslated regions (UTR), promotor/regulatory elements, and introns that are important in functional genomics studies. A BAC-based physical map is thus not just an interim solution or a step in the process of sequencing a genome but a viable resource for utilizing a complete genome sequence once ascertained. Physical maps have been reported recently for *Gossypium raimondii *[33], *Aquilegia formosa *[28], wheat chromosomes [34], maize [35,36], *Brachypodium distachyon *[37], *Oncorhynchus mykiss *[38], *Prunus persica *[39], and *Glycine max *[40,41].

Our *T. cacao *physical map assembly is highly ordered and tightly anchored to a moderately dense genetic recombination map, resides in 49 anchored contigs, and represents 82% of the *T. cacao *genome as computed by assuming a total genome size of 440 Mbp. However, estimated genome sizes for *T. cacao *range from 326 Mbp to 440 Mbp as determined using reassociation kinetics, flow cytometry [42,43], or genome assembly [11]. Once the true size of the genome of *T. cacao *cv. Matina 1-6 is known, our physical map statistics may require adjustment. Availability of a high-density genetic recombination map [5,26] was critical to our success in ordering and orienting BAC contigs and subsequently assigning contigs to chromosomes. We hybridized 230 genetically mapped SSR markers to the BAC contigs. Only 10 of these mapped markers hybridized to more than one contig signifying low copy-number, accuracy of the genetic map through additional evidence in the independent physical map build, and accuracy of the BAC assembly. Several of these markers flank QTL loci and therefore provide immediate templates for sequencing pools of high priority BACs to identify candidate genes for further investigation (companion paper). These mapped marker sequences also serve as sequence anchor points for use in comparative genome studies.

### Using other genomes to assess and improve the *T. cacao *physical map and discover biological insights

Each newly available genome assembly, especially those from species distantly related to model organisms, improves the resolution of genome biology and evolution. Exploration of evolving genome architecture through synteny analysis upon the release of a new genome has become a standard experiment in which a new window into a clade is opened. New genome assemblies are, however, incomplete. Gaps, chimeric joins, and unresolvable repeat-induced compressions are unavoidable. One way to identify these errors and improve an assembly is to use related genomes as a guide.

Comparative genomics has evolved rapidly over the last decade. More genomes acting as reference sequences and the advancement of computational algorithms and visualization software has facilitated a turnkey approach. At the same time, aligning physical maps to sequenced genomes has become increasingly useful. For example, Fang et al. constructed a physical map for *Aquilegia formosa*, a species in a unique clade of basal eudicots that is being utilized as a new model system for studying floral variation, adaptive radiation, and evolution, and used a comparative approach to gain insight into the evolutionary lineages between *A. formosa *and *V. vinifera *[28,44-46]. Gu et al. created a physical map of *Brachypodium distachyon *and compared it to rice and wheat; they observed whole-genome duplication events in relation to rice that were caused by paleotetraploidy and a broad spectrum of other evolutionary events between the wheat and *B. distachyon *genomes [37]. In short, a physical map serves as a distinct line of non-sequence-based evidence as well as an adjunct to a draft genome sequence during the biological discovery process.

In our comparative genome analysis, we used the SyMAP software to align the *T. cacao *cv. Matina 1-6 physical map to *A. thaliana, V. vinifera*, and *P. trichocarpa *genome sequences to gain insight into the structure and evolutionary history of *T. cacao *as well as to improve the physical map assembly. Not surprisingly, we found more syntenic blocks between the ten chromosomes of *T. cacao *and the 19 chromosomes of *P. trichocarpa *than between the ten chromosomes of *T. cacao *and the 19 chromosomes of grape. The main difference was in the length of the syntenic blocks; 100 syntenic blocks between *T. cacao *and *P. trichocarpa *were greater than 1 Mbp in length, whereas there were only 74 syntenic blocks longer than 1 Mbp between *T. cacao *and *V. vinifera*. The number of short (< 1 Mbp) collinear regions differed between these comparisons as well (27 in the *V. vinifera *comparison and 87 in the comparison with *P. trichocarpa*). Alignment to the *A. thaliana *genome produced the fewest syntenic blocks as expected since *A. thaliana *is the most evolutionarily distant species of the three used for our comparisons.

Structural and evolutionary implications can also be derived from comparisons between physical maps and available genome sequences. Collinear genomic segments and duplications can be quickly identified and provide insight into selective pressures and identify regions of the genome for targeted detailed analysis, all without a reference genome. As a result of our use of this approach, we concur that the chromosome fusion events recently reported by Argout et al. [11] occurred in the ancient genome structure that led to the ten-chromosome structure we see today. Additionally, this comparative approach is sufficient to identify and confirm ancestral triplicated chromosomal groups recently reported for *V. vinifera *[15].

### The utility of the cacao physical map with regard to cacao genome sequences

Even as more and more genomes are sequenced *de novo *and sequencing strategies evolve, *i.e*. there is less reliance on Sanger-based sequencing and second-generation sequencing platforms shift in chemistry and read-lengths, there are still inherent problems with assembly and resolution of distal regions of complex eukaryotic genomes. Mate-pair sequencing libraries with long insert sizes such as BACs and fosmids provide the necessary linking information and long-range contiguity for resolving repetitive DNA and scaffolding draft contigs. As noted previously, a physical map is an important adjunct to *de novo *genome sequencing projects and serves as an independent genome assembly that is composed of a very different data type. BAC fingerprints assembled (at a conservative cutoff) with an integrated dense array of paired-end sequences and genetic markers can be used to check for errors and to corroborate the accuracy of a draft genome assembled using a whole-genome sequencing (WGS) strategy. The physical map can also serve as a template for gap-filling and targeted sub-genome re-sequencing of BAC pools [47].

The physical map we present here can be of great utility in advancing draft genome sequences of *Theobroma cacao *into high quality reference genomes. For example, comparison of the cv. Matina 1-6 physical map assemblies to the cv. Criollo pseudomolecules revealed a high degree of collinear genomic segments (Figure 5A). However, there are still many regions of structural difference between these two draft sequences such as sequence inversions and translocations. There are also regions of discontiguous alignments between the physical map and draft sequence. We speculate that these differences may be the result of underlying biological differences between cultivars, misassemblies in either the Matina 1-6 physical map or the Criollo whole-genome draft assembly, gaps resulting from unresolved repetitive DNA, or a lack of dense BES in particular contigs in the physical map. For any of these possibilities, the Matina 1-6 physical map can serve as a template for confirming/resolving assembly differences and provide resolution in regions of low quality or underrepresentation in the draft genome assemblies through targeted PCR or sub-genome sequencing through BAC pools.

## Conclusions

The three BAC libraries, BAC-end sequences, and the genetically integrated physical map for *T. cacao *cv. Matina 1-6 resulting from this study are important resources for functional exploitation and enhancement of *Theobroma cacao *that are expected to complement and augment genome sequencing efforts. The results obtained from the comparative analyses with *A. thaliana, V. vinifera*, and *P. trichocarpa *suggest that genome assemblies from distantly related species can be used to gain insights into genome structure and evolutionary history as well as conserved functional genomic sites and to improve a physical map assembly. These resources will also serve as the templates for refinement of *T. cacao *genome sequences through gap-filling, targeted re-sequencing, and resolution of repetitive DNA arrays through long-range contiguity.

## Methods

### BAC library construction

The Matina 1-6 tree clones used for DNA isolation were kept at greenhouse conditions and dark-treated for 12 hours prior to leaf harvesting. Approximately 100 g of young, F2 stage expanding leaf tissue with the mid-vein removed were harvested, washed two times with autoclaved ddH_2_0 and ground in liquid nitrogen with a mortar and pestle to a coarse powder. Nuclei were prepared following previously published methods [24] with the following modifications: addition of 1% (w/v) soluble PVP-40 (Sigma-Aldrich), 0.1% (w/v) L-ascorbic acid (Sigma-Aldrich), 0.13% (w/v) sodium diethyldithiocarbamate trihydrate (Sigma-Aldrich), and 0.4% beta-mercaptoethanol to nuclei isolation buffer (NIB) right before use. Purified nuclei were concentrated and embedded in agarose plugs. Protein digestion and plug washing was carried out exactly as previously described [24]. To prepare high molecular weight genomic DNA fragments, plugs were macerated with a single-edge razor blade and then partially digested (separately) with, *Hin*dIII, *Eco*RI, or *Mbo*I using standard procedures. DNA size selection, electro-elution, and ligation were carried out as previously described [24]. The BAC libraries were assigned a unique CUGI identifier according the enzyme used for partial digestion: TCC_Ba was made using *Hin*dIII, TCC_Bb with *Mbo*I, and TCC_Bc with *Eco*RI. Each BAC library was characterized for insert distribution by randomly selecting 384 clones and subjecting them to miniprep, digestion with Not-I (New England Biolabs), and resolution by pulsed-field gel electrophoresis [23].

### HICF BAC fingerprinting

384-well plates containing BACs were decondensed to 96-well format robotically with the Q-bot (Genetix, United Kingdom). Two pins were removed from the sub-plate inoculators to allow for manual insertion of control clones in the E07 and H12 positions; controls were used to assess data uniformity. DNA was isolated from a total of 108,288 clones from TCC_Ba, TCC_Bb, and TCC_Bc, the three BAC libraries, by following standard alkaline lysis miniprep methods [48], and then used as substrates for HICF carried out following previously published methods [24] with the following modifications. Approximately 0.5 ug of BAC DNA was digested with 2.0 units of *Hin*dIII, *Bam*HI, *Xba*I, *Xho*I, and *Hae*III at 37C for 2 hours. The DNA was treated with 0.25 U of label from the SNaP-shot kit (Applied Biosystems, Foster City, California) at 65 C for 1 hour and then precipitated with ethanol. The labelled DNA was reconstituted in 9 ul of Hi-Di formamide (Applied Biosystems) and 0.05 ul of LIZ600 (Applied Biosystems). BAC fingerprints were sized on an ABI3730 (Applied Biosystems) using a 36 cm array and POP7 (Applied Biosystems). The fingerprint profiles were processed using GeneMapper 3.7 (Applied Biosystems) for sizing quality and FPMiner (Bioinforsoft, Oregon) for digitized fingerprint assignment. For improved data quality, vector bands, clones without inserts, and restriction profiles with less than 20 or more than 200 bands were removed and the remaining profiles were uploaded to FPC v8.5.3 [25] for fingerprint contig assembly.

### Physical map assembly

The initial build was done at a Sulston score cut-off of 1 e^-80 ^and a tolerance of 3. To reduce false joins, the DQer function of FPC was used to break down all contigs comprised of more than 10% questionable (Q) clones. Further physical map refinement was performed using the Ends-to-Ends and Singles-to-Ends functions of FPC with stepwise reductions of the Sulston score cutoff values to a final score of 1 e^-50^. Additional fingerprint contig merges were made with lower Ends-to-Ends overlap when there was additional agreement with the anchored genetic map [30]. Contigs merged in this fashion used Sulston score cutoffs as low as 1 e^-25^. High quality marker sequence was processed through a CUGI pipeline consisting of Repeat Masker [49] with the RepBase database [50], cross_match [51], and Tandem Repeat Finder [52]. The remaining sequence was used for overgo design using the overgomaker (http://genome.wustl.edu/software/overgo_maker) software. Once a version of the whole-genome draft sequence of Matina 1-6 became available [12], potentially repetitive overgo sequences were aligned using BLAST [53] with cutoff of at least 85% percent identity. Any overgo sequence with more than 8 hits to the putative assembly was removed from the experiment, the repetitive sequence was masked, and a new overgo was selected from the original sequence. Manual filter hit-calling and deconvolution of the multi-dimensional pool hybridization data was accomplished using HybDecon, open source software available at http://www.genome.clemson.edu/software/hybdecon. The filter hit-calling functionality is an improved version of Hybsweeper, a web-based Java tool first reported by Lazo et al. [54]. In addition, a Perl-based deconvolution script, written entirely at CUGI, accompanies the tool and is launched from within the graphical interface once manual calling of positive hits is complete. The source code, a test dataset, and installation manual are all available online. A user's manual is readily available with the click of the Help button from within the software. Additionally an online experimental design tool is available (http://www.genome.clemson.edu/software/hybdecon/exp_setup) to assist with setup for multi-dimensional pooling hybridization.

### Dense MTP selection and BAC-end sequencing

We selected a set of BACs from the physical map to provide sequenced BAC-ends at least every 8 kb along the genomic sequence. However, because the unit of measure in an FPC physical map is a Consensus Band (CB) unit, or restriction fragment unit, which does not correlate with distance in base pairs, we estimated the number of CB units that would approximate a distance of 20 kb using the following formula:

$$n=\frac{m}{i\;/)\;k}$$

where m is median number of CB units per BAC in the physical map, i is the average BAC insert size, k is the desired size in base pairs, and n is the number of CB units that estimates a distance of 20 kb. An in-house Perl script was created to iterate through the contigs of the physical map and select BACs which were closest to being 7 CB units apart. A list of BAC-ends that existed prior to this analysis was provided as input to the script and these BACs were used to avoid selection of a new BAC in regions where BAC-ends already existed. BACs were selected first by the CB location of their left-ends, but new BACs were not selected in regions where right-ends from a previous selection existed.

### Pseudomolecule construction and genome size estimation

Physical map coordinates for markers (CB position, contig ID) were obtained directly from FPC by right-clicking 'file' and selecting 'save ordered marker list'. Contig lengths and BAC positions were manually parsed from FPC using the following procedure: A) 'cat < fpc_filename > | grep 'Map' | sed s/Map//| sed s/Ends//| sed s/ctg//| cut -d "O" -f1 | cut -d "." -f1 > BacsInContigs.txt'; B) BacsInContigs.txt was dropped into Excel and split at space delimiters and parsed; C) BAC start and stop positions in a contig and total BAC counts per contig was determined using a 'find duplicates' query in Access 2010 (Microsoft). Contigs that contained a genetic marker were considered anchored and assigned to a linkage group based upon that marker. Marker order within a contig was determined based upon physical position and not genetic position. Ordered and anchored contigs were then assigned physical map positions. Anchored contigs were sorted according to genetic position and pseudomolecules were constructed by adding an arbitrary gap of 0.25 Mbp between anchored contigs. Finally, a CMAP file was constructed for 10 anchored linkage group maps (Pseudo1-Pseudo10) with the following four feature types: contig, gap, marker, and STS. Genome size was estimated as the sum of anchored contigs (+ gaps) and unanchored contigs.

### Synteny mapping and comparative genomics

Synteny mapping was carried out using the Symap software [31,32]. Genomic assemblies and annotations for alignments were downloaded from the following locations: *A. thaliana *(http://www.arabidopsis.org/portals/genAnnotation/gene_structural_annotation/agicomplete.jsp), *V. vinifera *(http://www.plantgdb.org/VvGDB/), *P. trichocarpa *(http://www.phytozome.net/poplar.php), and *T. cacao *cv. Criollo (http://cocoagendb.cirad.fr). The *T. cacao *cv. Matina 1-6 input data for Symap was our 49 BAC contigs anchored to chromosomes by 230 genetic recombination markers and aligned to the respective genomes with 52,966 BAC-end sequences and the 230 genetic map sequences, respectively.

## Authors' contributions

CAS drafted the manuscript, constructed BAC libraries, performed FPC and comparative analysis; FAF assisted with manuscript preparation, performed pseudomolecule construction, and critically reviewed all data. MES created cMAP presentation and renamed sequences. BPB performed HICF fingerprinting and initial FPC analysis. SPF selected dense array of BACs for end sequencing and performed sequence trimming. DNK, RS, and JCM conceived and implemented the study and reviewed the manuscript. All authors have read and approved the final manuscript.

## Supplementary Material

Additional file 1 **Genetic marker BAC hybridizations**. Summary of anchoring the Matina 1-6 genetic map to the Matina 1-6 physical map. Click here for file

Additional file 2 **Integration of genetic map to physical map through overgo probe hybridization**. Genetic markers, linkage groups, and respective recombination distances. Click here for file

Additional file 3 **Genetically anchored FPC contigs**. Assembled physical map contigs that were genetically anchored, with corresponding lengths and number of BACs. Click here for file

Additional file 4 **Unanchored FPC contigs**. Assembled physical map contigs that were not genetically anchored, with corresponding lengths and number of BACs. Click here for file

Additional file 5 ***T. cacao *physical map BES aligned to *V. vinifera genome assembly***. Alignment of the Matina 1-6 physical map to the *Vitis *genome. Click here for file

Additional file 6 ***T. cacao *physical map BES aligned to *P. trichocarpa *genome assembly**. Alignment of the Matina 1-6 physical map to the Populus genome. Click here for file

Additional file 7 ***T. cacao *physical map BES aligned to *A. thaliana *genome assembly**. Alignment of the Matina 1-6 physical map to the Arabidopsis genome. Click here for file

Additional file 8 ***T. cacao *physical map BES aligned to *T. cacao *(Criollo) genome assembly**. Alignment of the Matina 1-6 physical map to the Criollo genome. Click here for file

Additional file 9 **LG prediction of unanchored FPC contigs based on collinearity with other genomes**. Summary of incorporation of unanchored contigs. Click here for file

## References

[B1] Evans HC Cacao diseases-the trilogy revisited Phytopath 2007 97 12 1640 1643 10.1094/PHYTO-97-12-1640 18943725

[B2] Hebbar PK Cacao diseases: a global perspective from an industry point of view Phytopath 2007 97 12 1658 1663 10.1094/PHYTO-97-12-1658 18943730

[B3] Brown JS Phillips-Mora W Power EJ Krol C Cervantes-Martinez C Motamayor JC Schnell RJ Mapping QTLs for Resistance to Frosty Pod and Black Pod Diseases and Horticultural Traits in Theobroma cacao Crop Science 2007 47 5 1851 1858 10.2135/cropsci2006.11.0753

[B4] Clement D Risterucci AM Motamayor JC N'Goran J Lanaud C Mapping QTL for yield components, vigor, and resistance to Phytophthora palmivora in Theobroma cacao L Genome 2003 46 2 204 212 10.1139/g02-125 12723036

[B5] Crouzillat D Lerceteau E Pétiard V Morera-Monge JA Rodríguez H Walker D Phillips-Mora W Ronning C Schnell RJ Osei J Theobroma cacao L.: A genetic linkage map and quantitative trait loci analysis Theor Appl Genet 1996 93 1-2 205 214 10.1007/BF00225747 24162219

[B6] Faleiro F Queiroz V Lopes U Guimarães C Pires J Yamada M Araújo I Pereira M Schnell R Filho G Mapping QTLs for Witches' Broom (Crinipellis Perniciosa) Resistance in Cacao (Theobroma Cacao L.) Euphytica 1996 149 1-2 227 235

[B7] Lanaud C Fouet O Clément D Boccara M Risterucci AM Surujdeo-Maharaj S Legavre T Argout X A meta-QTL analysis of disease resistance traits of Theobroma cacao L Molecular Breeding 2009 24 4 361 374 10.1007/s11032-009-9297-4

[B8] Queiroz VT Guimarães CT Anhert D Schuster I Daher RT Pereira MG Miranda VRM Loguercio LL Barros EG Moreira MA Identification of a major QTL in cocoa (Theobroma cacao L.) associated with resistance to witches' broom disease Plant Breeding 2003 122 3 268 272 10.1046/j.1439-0523.2003.00809.x

[B9] Risterucci AM Paulin D Ducamp M N'Goran JA Lanaud C Identification of QTLs related to cocoa resistance to three species of Phytophthora Theor Appl Genet 2003 108 1 168 174 10.1007/s00122-003-1408-8 13679987

[B10] Schnell RJ Kuhn DN Brown JS Olano CT Phillips-Mora W Amores FM Motamayor JC Development of a marker assisted selection program for cacao Phytopath 2007 97 12 1664 1669 10.1094/PHYTO-97-12-1664 18943731

[B11] Argout X Salse J Aury JM Guiltinan MJ Droc G Gouzy J Allegre M Chaparro C Legavre T Maximova SN The genome of Theobroma cacao Nature Genetics 2011 43 2 101 108 10.1038/ng.736 21186351

[B12] http://www.cacaogenomedb.org

[B13] IRGSP The map-based sequence of the rice genome Nature 2005 436 7052 793 800 10.1038/nature03895 16100779

[B14] Pennisi E Plant sciences. Corn genomics pops wide open Science 2008 319 5868 1333 10.1126/science.319.5868.1333 18323430

[B15] Jaillon O Aury JM Noel B Policriti A Clepet C Casagrande A Choisne N Aubourg S Vitulo N Jubin C The grapevine genome sequence suggests ancestral hexaploidization in major angiosperm phyla Nature 2007 449 7161 463 467 10.1038/nature06148 17721507

[B16] Paterson AH Bowers JE Bruggmann R Dubchak I Grimwood J Gundlach H Haberer G Hellsten U Mitros T Poliakov A The Sorghum bicolor genome and the diversification of grasses Nature 2009 457 7229 551 556 10.1038/nature07723 19189423

[B17] Tuskan GA Difazio S Jansson S Bohlmann J Grigoriev I Hellsten U Putnam N Ralph S Rombauts S Salamov A The genome of black cottonwood, Populus trichocarpa (Torr. & Gray) Science 2006 313 5793 1596 1604 10.1126/science.1128691 16973872

[B18] Nelson W Soderlund C Integrating sequence with FPC fingerprint maps Nucleic Acids Res 2009 37 5 e36 10.1093/nar/gkp034 19181701 PMC2655663

[B19] van Oeveren J de Ruiter M Jesse T van der Poel H Tang J Yalcin F Janssen A Volpin H Stormo KE Bogden R Sequence-based physical mapping of complex genomes by whole genome profiling Genome Res 2011 10.1101/gr.112094.110 PMC3065709 21324881

[B20] Zhu H Blackmon BP Sasinowski M Dean RA Physical map and organization of chromosome 7 in the rice blast fungus, Magnaporthe grisea Genome Res 1999 9 8 739 750 10447509 PMC310806

[B21] Soderlund C Longden I Mott R FPC: a system for building contigs from restriction fingerprinted clones Comput Appl Biosci 1997 13 5 523 535 9367125 10.1093/bioinformatics/13.5.523

[B22] Xiong Z Kim JS Pires JC Integration of genetic, physical, and cytogenetic maps for Brassica rapa chromosome A7 Cytogenet Genome Res 2010 129 1-3 190 198 10.1159/000314640 20628251

[B23] Luo M Wing RA An Improved Method for Plant BAC Library Construction 2003 236 Totowa, NJ: Humana Press, Inc 10.1385/1-59259-413-1:3 14501055

[B24] Luo MC Thomas C You FM Hsiao J Ouyang S Buell CR Malandro M McGuire PE Anderson OD Dvorak J High-throughput fingerprinting of bacterial artificial chromosomes using the snapshot labeling kit and sizing of restriction fragments by capillary electrophoresis Genomics 2003 82 3 378 389 10.1016/S0888-7543(03)00128-9 12906862

[B25] Soderlund C Humphray S Dunham A French L Contigs built with fingerprints, markers, and FPC V4.7 Genome Res 2000 10 11 1772 1787 10.1101/gr.GR-1375R 11076862 PMC310962

[B26] Brown JS Schnell RJ Motamayor JC Lopes U Kuhn DN Borrone JW Resistance gene mapping for witches' broom disease in Theobroma cacao L. in an F2 population using SSR markers and candidate genes J Amer Soc Hort Sci 2005 130 3 366 373

[B27] Wu F Mueller LA Crouzillat D Petiard V Tanksley SD Combining bioinformatics and phylogenetics to identify large sets of single-copy orthologous genes (COSII) for comparative, evolutionary and systematic studies: a test case in the euasterid plant clade Genetics 2006 174 3 1407 1420 10.1534/genetics.106.062455 16951058 PMC1667096

[B28] Fang GC Blackmon BP Henry DC Staton ME Saski CA Hodges SA Tomkins JP Luo H Genomic tools development for Aquilegia: construction of a BAC-based physical map BMC Genomics 2010 11 621 10.1186/1471-2164-11-621 21059242 PMC3091760

[B29] Fang Z Polacco M Chen S Schroeder S Hancock D Sanchez H Coe E cMap: the comparative genetic map viewer Bioinformatics 2003 19 3 416 417 10.1093/bioinformatics/btg012 12584129

[B30] Brown JS Sautter RT Olano CT Borrone JW Kuhn DN Motamayor JC Schnell RJ A Composite Linkage Map from Three Crosses Between Commercial Clones of Cacao, Theobroma cacao L Tropical Plant Biol 2008 1 2 120 130 10.1007/s12042-008-9011-4

[B31] Soderlund C Nelson W Shoemaker A Paterson A SyMAP: A system for discovering and viewing syntenic regions of FPC maps Genome Res 2006 16 9 1159 1168 10.1101/gr.5396706 16951135 PMC1557773

[B32] Soderlund C Bomhoff M Nelson WM SyMAP v3.4: a turnkey synteny system with application to plant genomes Nucleic Acids Res 2011 10.1093/nar/gkr123 PMC3105427 21398631

[B33] Lin L Pierce GJ Bowers JE Estill JC Compton RO Rainville LK Kim C Lemke C Rong J Tang H A draft physical map of a D-genome cotton species (Gossypium raimondii) BMC Genomics 2010 11 395 10.1186/1471-2164-11-395 20569427 PMC2996926

[B34] Luo MC Ma Y You FM Anderson OD Kopecky D Simkova H Safar J Dolezel J Gill B McGuire PE Feasibility of physical map construction from fingerprinted bacterial artificial chromosome libraries of polyploid plant species BMC Genomics 2010 11 122 10.1186/1471-2164-11-122 20170511 PMC2836288

[B35] Schnable PS Ware D Fulton RS Stein JC Wei F Pasternak S Liang C Zhang J Fulton L Graves TA The B73 maize genome: complexity, diversity, and dynamics Science 2009 326 5956 1112 1115 10.1126/science.1178534 19965430

[B36] Wei F Zhang J Zhou S He R Schaeffer M Collura K Kudrna D Faga BP Wissotski M Golser W The physical and genetic framework of the maize B73 genome PLoS Genet 2009 5 11 e1000715 10.1371/journal.pgen.1000715 19936061 PMC2774505

[B37] Gu YQ Ma Y Huo N Vogel JP You FM Lazo GR Nelson WM Soderlund C Dvorak J Anderson OD A BAC-based physical map of Brachypodium distachyon and its comparative analysis with rice and wheat BMC Genomics 2009 10 496 10.1186/1471-2164-10-496 19860896 PMC2774330

[B38] Palti Y Luo MC Hu Y Genet C You FM Vallejo RL Thorgaard GH Wheeler PA Rexroad CE A first generation BAC-based physical map of the rainbow trout genome BMC Genomics 2009 10 462 10.1186/1471-2164-10-462 19814815 PMC2763887

[B39] Zhebentyayeva T Swire-Clark G Georgi L Garay L Jung S Forrest S Blenda A Blackmon B Mook J Horn R A framework physical map for peach, a model Rosaceae species 2008 4 745 756

[B40] Schmutz J Cannon SB Schlueter J Ma J Mitros T Nelson W Hyten DL Song Q Thelen JJ Cheng J Genome sequence of the palaeopolyploid soybean Nature 2010 463 7278 178 183 10.1038/nature08670 20075913

[B41] Wu C Sun S Nimmakayala P Santos FA Meksem K Springman R Ding K Lightfoot DA Zhang HB A BAC- and BIBAC-based physical map of the soybean genome Genome Res 2004 14 2 319 326 10.1101/gr.1405004 14718376 PMC327108

[B42] Figueira A Janick J Goldsbrough P Genome Size and DNA Polymorphism in Theobroma-Cacao J Amer Soc Hort Sci 1992 117 4 673 677

[B43] Couch JA Zintel HA Fritz PJ The genome of the tropical tree Theobroma cacao L Mol Gen Genet 1993 238 123 128 10.1007/BF00282792 8455550

[B44] Kramer EM Aquilegia: A New Model for Plant Development, Ecology, and Evolution Annual Review of Plant Biology 2009 60 261 277 10.1146/annurev.arplant.043008.092051 19575583

[B45] Kramer EM Hodges SA Aquilegia as a model system for the evolution and ecology of petals Phil Trans R Soc B 2010 365 477 490 10.1098/rstb.2009.0230 20047874 PMC2838260

[B46] Hodges SA Derieg NJ Adaptive radiations: From field to genomic studies Proc Natl Acad Sci USA 2009 106 9947 9954 10.1073/pnas.0901594106 19528644 PMC2702792

[B47] Rounsley S Marri PR Yu Y He R Sisneros N Goicoechea JL Lee SJ Angelova A Kudrna D Luo M De Novo Next Generation Sequencing of Plant Genomes Rice 2009 2 1 1939 8425

[B48] Sambrook J Fitsch EF Maniatis T Molecular Cloning: A Laboratory Manual 1989 Cold Spring Harbor, NY: Cold Spring Harbor Press

[B49] RepeatMasker http://www.repeatmasker.org

[B50] Jurka J Kapitonov VV Pavlicek A Klonowski P Kohany O Walichiewicz J Repbase Update, a database of eukaryotic repetitive elements Cytogenet Genome Res 2005 110 1-4 462 467 10.1159/000084979 16093699

[B51] Gordon D Abajian C Green P Consed: a graphical tool for sequence finishing Genome Res 1998 8 3 195 202 9521923 10.1101/gr.8.3.195

[B52] Benson G Tandem repeats finder: a program to analyze DNA sequences Nucleic Acids Res 1999 27 2 573 580 10.1093/nar/27.2.573 9862982 PMC148217

[B53] Altschul SF Madden TL Schaffer AA Zhang J Zhang Z Miller W Lipman DJ Gapped BLAST and PSI-BLAST: a new generation of protein database search programs Nucleic Acids Res 1997 25 17 3389 3402 10.1093/nar/25.17.3389 9254694 PMC146917

[B54] Lazo GR Lui N Gu YQ Kong X Coleman-Derr D Anderson OD Hybsweeper: a resource for detecting high-density plate gridding coordinates Biotechniques 2005 39 3 320 322, 324 10.2144/05393BM05 16206904

